# Synthesis, crystal structure and Hirshfeld surface analysis of 4-(3-hy­droxy-6-meth­oxy-4-oxo-4*H*-chromen-2-yl)benzaldehyde

**DOI:** 10.1107/S2056989026003476

**Published:** 2026-04-10

**Authors:** Arsenii D. Snizhko, Viktoriya V. Dyakonenko, Eugene S. Gladkov, Alexander V. Kyrychenko

**Affiliations:** a V. N. Karazin Kharkiv National University, 4 Svobody Sq., Kharkiv 61022, Ukraine; b Institute of Functional Materials Chemistry, SSI "Institute for Single Crystals" of National Academy of Sciences of Ukraine, Nauki Ave 60, Kharkiv, 61001, Ukraine; c V. I. Vernadskii Institute of General and Inorganic Chemistry of National Academy of Sciences of Ukraine Prospect Palladina 32/34 03680 Kyiv Ukraine; Venezuelan Institute of Scientific Research, Venezuela

**Keywords:** crystal structure, flavonol derivatives, chromone, ESIPT (Excited-State Intra­molecular Proton Transfer), Hirshfeld surface analysis

## Abstract

The asymmetric unit of the title compound, C_17_H_12_O_5_, contains one crystallographically independent mol­ecule featuring a chromenone fragment with hy­droxy and meth­oxy substituents and a benzaldehyde group. Intra­molecular O—H⋯O, C—H⋯O hydrogen bonds are observed. In the crystal, mol­ecules are linked by O—H⋯O inter­molecular bonds, forming chains along [201]. The Hirshfeld surface analysis shows that the H⋯H and O⋯H/H⋯O contacts dominate the crystal packing with contributions of 34.2% and 27.6%, respectively.

## Abstract 2

The asymmetric unit of the title compound contains one crystallographically independent mol­ecule featuring a chromenone fragment with hy­droxy and meth­oxy substituents and a benzaldehyde group. Intra­molecular O—H⋯O, C—H⋯O hydrogen bonds are observed. In the crystal, mol­ecules are linked by O—H⋯O inter­molecular bonds, forming chains along [201].

## 1. Chemical context

Compounds bearing a 3-hy­droxy-2-phenyl-chromen-4-one core belong to the family specifically categorized as flavonol derivatives (common name: 3-hy­droxy­flavones). They feature a chromone (1-benzo­pyran-4-one) core, which is a privileged scaffold in medicinal chemistry due to its diverse biological activities (Borsari *et al.*, 2016 ▸). These derivatives, also referred to as 3-hy­droxy­flavones, can scavenge free radicals and chelate metal ions (Roshal, 2024 ▸; Mihajlović *et al.*, 2025 ▸), which is vital in preventing oxidative stress-related diseases.

In 3-hy­droxy­flavone derivatives, a crucial intra­molecular hydrogen bond forms between the 3-hydroxyl group and the 4-oxo (carbonyl, C=O) oxygen. This bond forms a five-membered ring stabilized by a resonance-assisted hydrogen bond and is responsible for excited-state intra­molecular proton transfer (ESIPT) (Zhao *et al.*, 2021 ▸; Pivovarenko, 2023 ▸; Pivovarenko & Klymchenko, 2024 ▸). The mol­ecule absorbs light in its enol form but, after the proton jumps, it emits light as a keto form. This keto form has a much lower energy, shifting the emission to much longer wavelengths. ESIPT features can be tuned by C4′ substitutions making it possible to use 3-hy­droxy­flavones as environment-sensitive fluorescence probes (Pivovarenko, 2023 ▸; Snizhko *et al.*, 2025 ▸; Chepeleva *et al.*, 2023 ▸; Demidov *et al.*, 2022 ▸; Kyrychenko & Ladokhin, 2024 ▸).

The X-ray structures were vital for showing how various electron-donating and electron-withdrawing groups, especially at the C6, C7, and C4′ positions, and steric factors affect the planarity of 3-hy­droxy­flavones (Etter *et al.*, 1986 ▸; Shoja *et al.*, 1998 ▸; Shoja & Sullivan, 1999 ▸; Wera *et al.*, 2011*a* ▸,*b* ▸; Narita *et al.*, 2015 ▸; Koh, 2020 ▸). Recently, we have demonstrated that the nature and position of substituent groups can significantly influence crystal packing in the solid state, thereby tuning the contributions of intra- and inter­molecular hydrogen bonding and the ESIPT behavior (Demidov *et al.*, 2025 ▸). The investigation of the crystal structure of 3-hy­droxy­flavones bearing a C6-electron-donating group on the *A* ring and a C4′-electron-withdrawing group on the *B* ring provides insights into the role of electron conjugation and push–pull effects (Pivovarenko & Klymchenko, 2024 ▸; Doroshenko *et al.*, 2019 ▸, 2026 ▸) on the structure, optical properties and supra­molecular inter­actions.
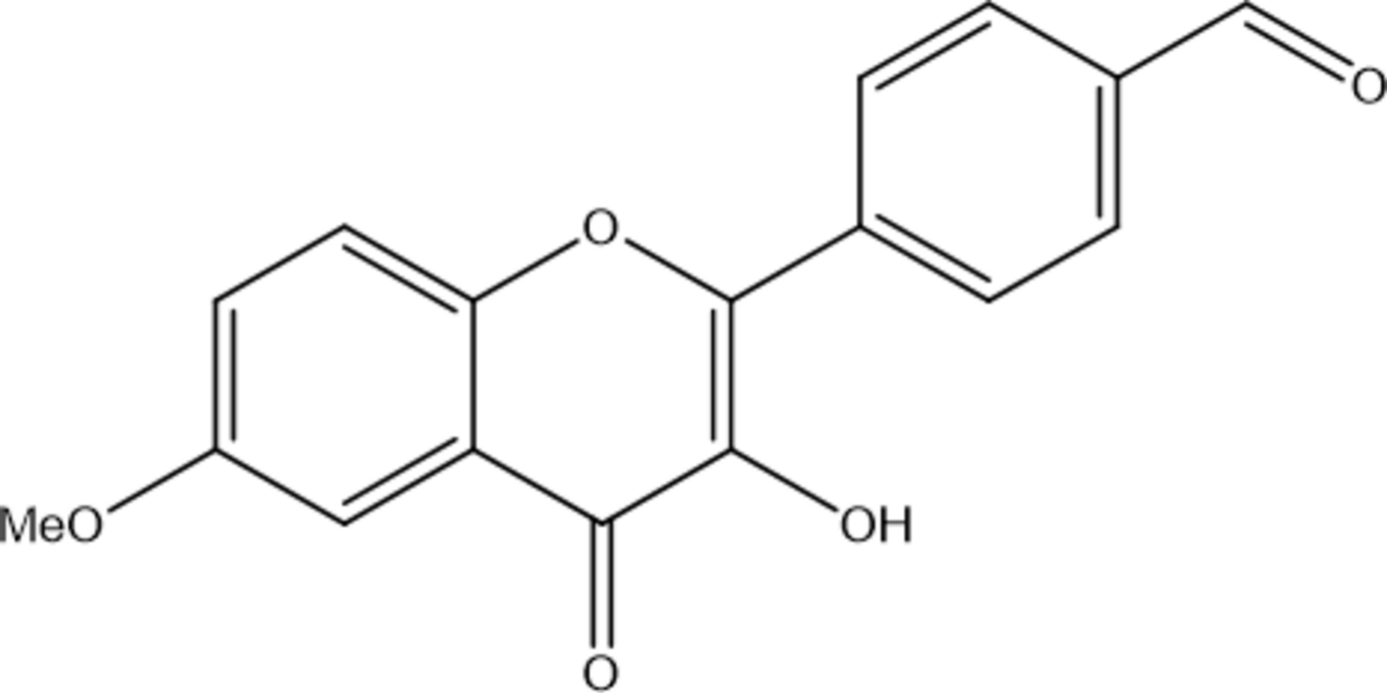


## 2. Structural commentary

**Figure 1:**
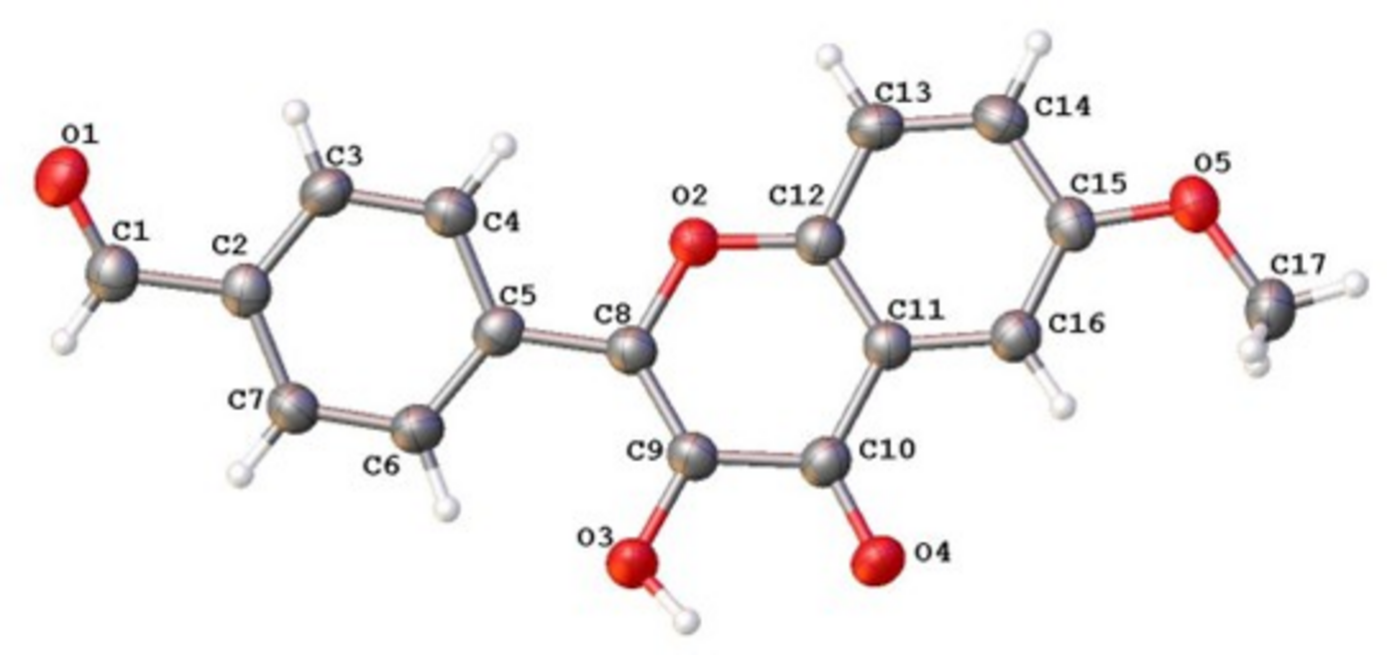
The mol­ecular structure of **1**, showing the atom labeling and displacement ellipsoids drawn at the 50% probability level.

The mol­ecular structure of the title compound, **1**, is shown in Fig. 1 ▸. The asymmetric unit contains one crystallographically independent mol­ecule. The mol­ecule comprises a chromenone fragment bearing hy­droxy and meth­oxy substituents and a benzaldehyde group. The meth­oxy substituent at atom C15 is almost coplanar with the chromenone fragment, as indicated by the C17—O5—C15—C16 torsion angle of 3.4 (3)°.

The benzaldehyde ring is rotated relative to the chromenone fragment. The inter­fragment torsion angle C6—C5—C8—C9 is −12.8 (3)°, indicating a slight twist between the two fragments. This orientation enables an intra­molecular C6—H6⋯O3 hydrogen bond (Table 1 ▸) involving a phenyl C—H group and an oxygen atom of the chromenone moiety. The oxygen atom O3, which participates in the intra­molecular C—H⋯O inter­action also participates in the intra­molecular resonant O3—H3⋯O4 hydrogen bond in the chromenone group (Table 1 ▸). This hydrogen bond is important as it is responsible for ESIPT in compounds of this type, as mentioned earlier in the *Chemical context*. Thus, the O3 atom participates in two intra­molecular hydrogen bonds of different types.

## 3. Supra­molecular features

**Figure 2:**
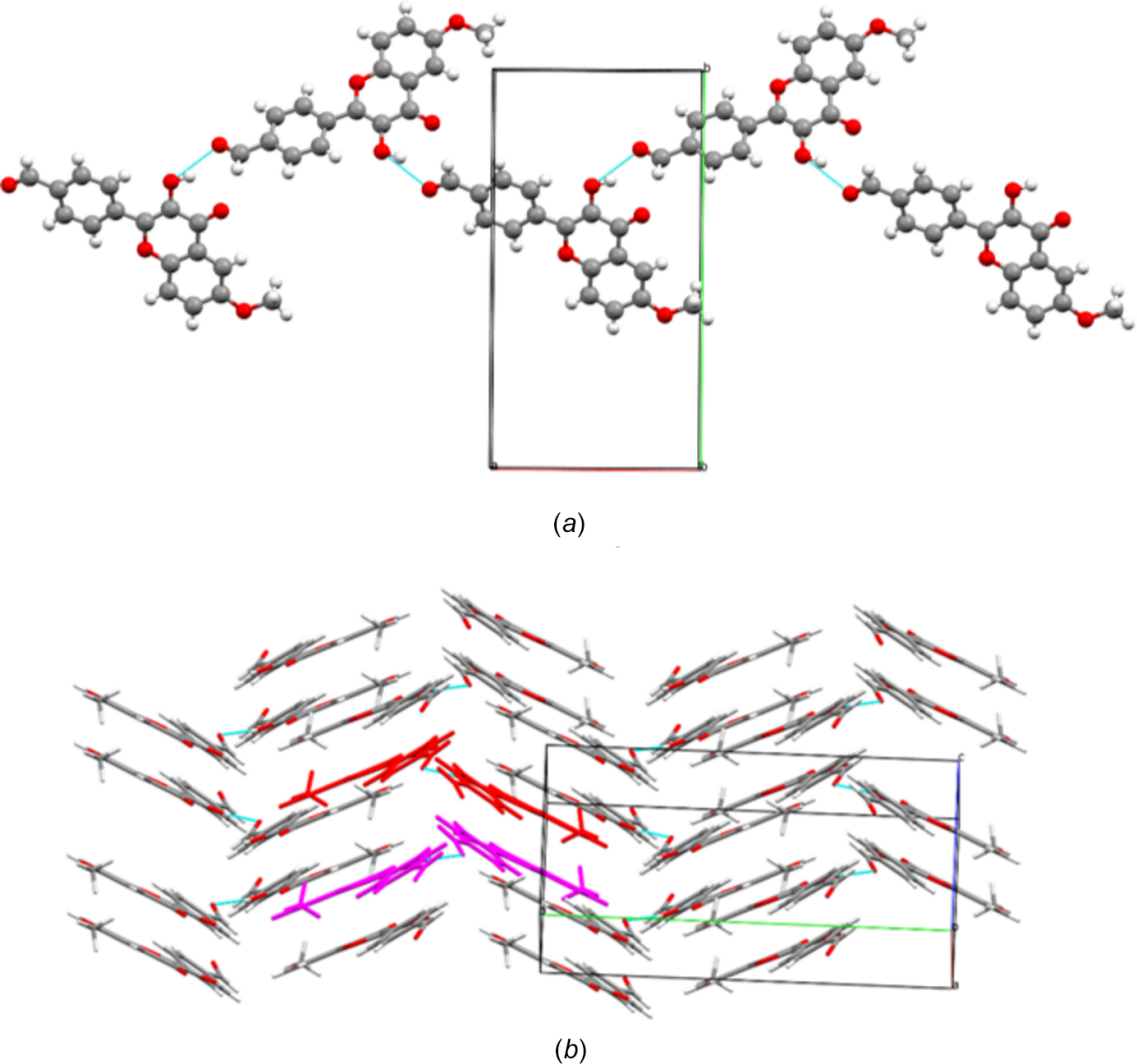
(*a*) The hydrogen-bonded chains of **1**. (*b*) The crystal packing of **1**. Some hydrogen-bonded chains are highlighted in different colors.

In the crystal, mol­ecules of **1** are linked by O3—H3⋯O1^i^ hydrogen bonds (Table 1 ▸, Fig. 2 ▸*a*), forming zigzag chains along [201] (Fig. 2 ▸*b*). The crystal packing is further consolidated by π–π stacking inter­actions between chromenone rings of mol­ecules belonging to adjacent chains [*Cg*1⋯*Cg*3(1 − *x*, 1 − *y*, 1 − *z*) = 3.575 (3) Å; *Cg*1 and *Cg*3 are the centroids of the O2/C8–C12 and C11 –C16 rings, respectively]. In addition, weak C7—H7⋯C3(*x*, 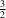 − *y*, −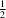 + *z*)(π) contacts are observed between mol­ecules from neighboring chains [H⋯C = 2.86 (3) Å, C—H⋯C = 170.4 (2)°], which also contribute to the cohesion of the crystal packing.

## 4. Hirshfeld surface analysis and fingerprint plots

**Figure 3:**
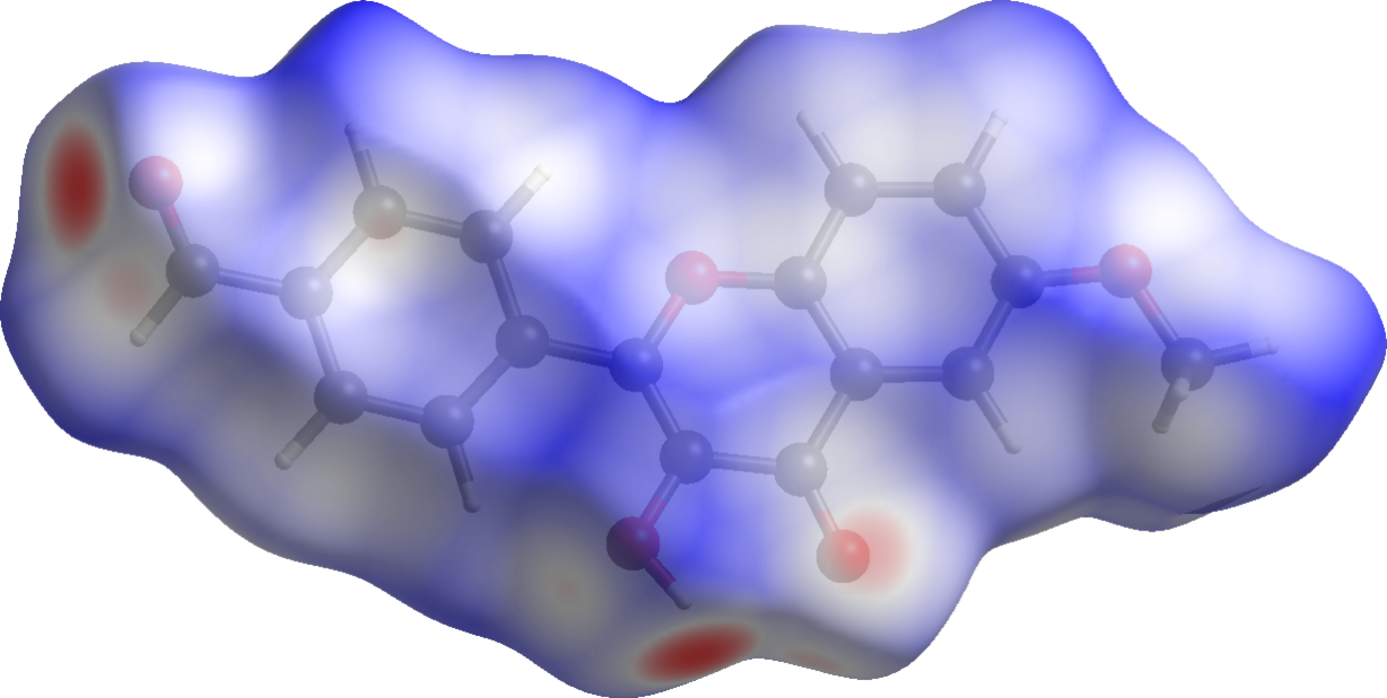
Three-dimensional Hirshfeld surface of title compound mapped over *d*_norm_.

The inter­molecular inter­actions were visualized using the *CrystalExplorer21* program (Spackman *et al.*, 2021 ▸). The Hirshfeld surface mapped over *d*_norm_ (Spackman & Jayatilaka, 2009 ▸) is shown in Fig. 3 ▸. The strongest contacts, which are visualized on the Hirshfeld surface as the dark-red spots, correspond to the inter­molecular O—H⋯O hydrogen bond between mol­ecules. Lighter red spots correspond to weaker O⋯H/H⋯O inter­actions, such as C—H⋯O. The majority of the inter­molecular inter­actions of **1** are weak, which results in the blue color of the Hirshfeld surface.

**Figure 4:**
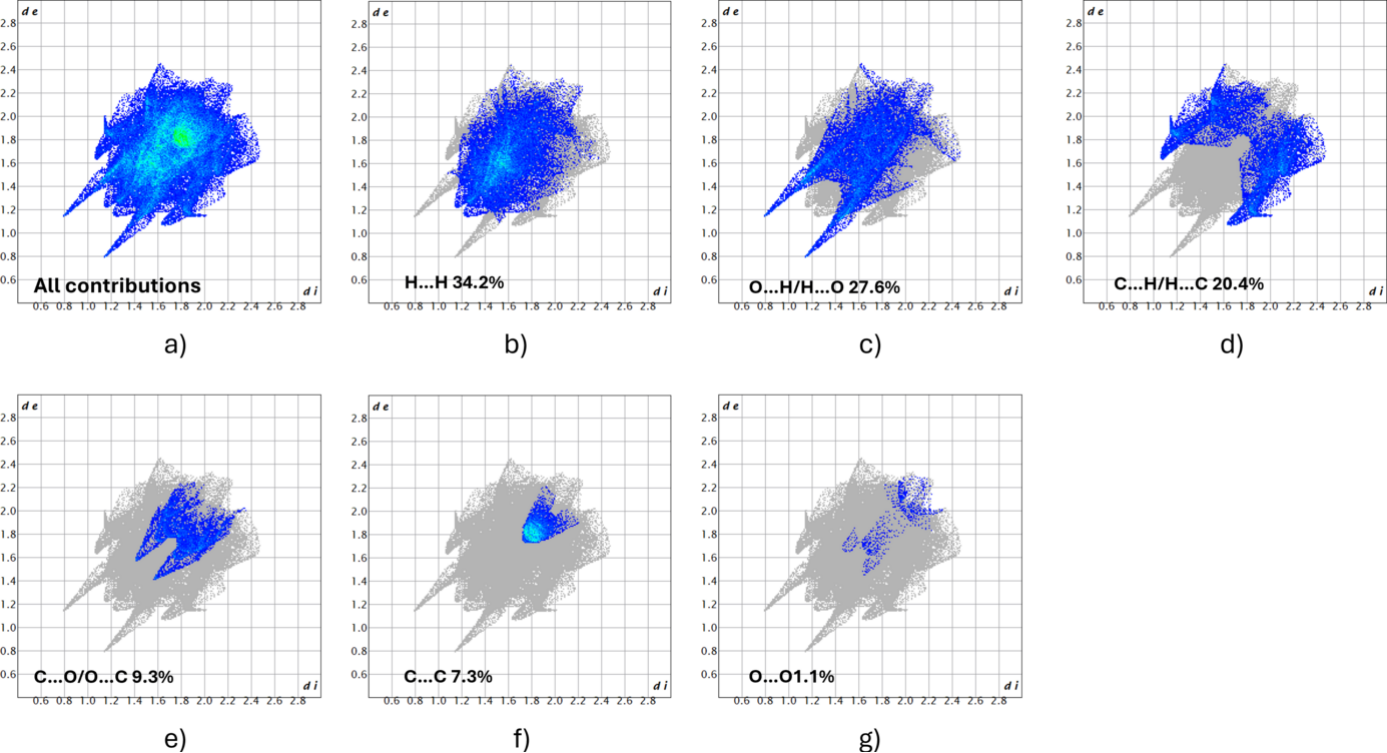
Two-dimensional fingerprint plots for the title compound showing (*a*) all inter­actions, and (*b*)–(*g*) delineated into contributions from other contacts [*d*_e_ and *d*_i_ represent the distances from a point on the Hirshfeld surface to the nearest atoms outside (external) and inside (inter­nal) the surface, respectively].

For further exploration of the inter­molecular inter­actions, two-dimensional fingerprint plots (McKinnon *et al.*, 2007 ▸) were generated as shown in Fig. 4 ▸. The H⋯H and O⋯H/H⋯O inter­actions, with contributions of 34.2% and 27.6%, respectively, have the greatest impact on the crystal packing in the solid state. The C⋯H/H⋯C inter­actions with 20.4%, C⋯O/O⋯C with 9.3%, C⋯C with 7.3% or O⋯O with 1.1% are less impactful in comparison.

## 5. Database survey

A search of the Cambridge Structural Database (CSD, Version 6.00, updated March 2025; Groom *et al.*, 2016 ▸) found 74 structures containing 3-hydroxyflavone. Of these, we would like to highlight 13 hits that are similar to title compound. These hits include the parent 3-hy­droxy­flavone itself and its derivatives (refcodes DUMFAS, DUMFEW, DUMFIA; Etter *et al.*, 1986 ▸). The C4′-fluoro (WACTUR; Wera *et al.*, 2010 ▸), C4′-hy­droxy (IJUCAS; Wera *et al.*, 2011*a* ▸), and C4′-meth­oxy (IKAHIM; Wera *et al.*, 2011*b* ▸; IKAHIM01; Demidov *et al.*, 2025 ▸) derivatives have been reported. The C4′-*tert*-butyl 3-hydroxyflavone has been reported (OHELE; Narita *et al.*, 2015 ▸). Polymorphs of C4′-(di­methyl­amino) and C4′-(di­ethyl­amino) 3-hydroxyflavone has been found (BANJEN, BANJEN01, CEZDOC, CEZDOC; Hino *et al.*, 2011 ▸, 2013 ▸). 3-Hydroxyflavones with C2′-meth­oxy (LIGZIK01,; Shoja & Sullivan, 1999 ▸), C2′,C3-dimeth­oxy (PUWCUI; Koh, 2020 ▸), C3′-benz­oxy (AMEBAZ, AMORUT; Demidov *et al.*, 2025 ▸), and C7-meth­oxy (NUZPUT; Shoja *et al.*, 1998 ▸) groups have been reported.

## 6. Synthesis and crystallization

The compound was synthesized by a modified procedure reported earlier (Demidov *et al.*, 2025 ▸). All chemicals were purchased from commercial suppliers and used without further purification (Sigma-Aldrich, Enamine Ltd).

Hy­droxy-5-meth­oxy-aceto­phenone (1.66 g, 10 mmol) and terephthalaldehyde diethyl-acetal (2.08 g, 10 mmol) were dissolved in ethanol (40 mL). Potassium hydroxide (3.36 g, 60 mmol) was added to the solution under stirring at room temperature. The reaction mixture was stirred for 24 h and conversion was monitored by TLC. After completion of the reaction hydrogen peroxide (30% H_2_O_2_, 3.4 mL, 30 mmol) was added dropwise to the reaction mixture, which was then placed in the ultrasound bath at room temperature for 10 minutes. After that the mixture was cooled to 273 K and acidified with 10% hydro­chloric acid to reach a pH of 3 and stirred for additional 10 minutes. The resulting precipitate was filtered off and washed thoroughly with water and hexane. The crude product was recrystallized twice from *i*-PrOH–DMF (45:1) mixture. Yield 1.35 g (46%), yellow crystalline material, m.p. 472.5–473 K. Elemental analysis calculated for C_17_H_12_O_5:_ C, 68.92; H, 4.08. Found: C, 68.78; H, 4.15.

^1^H NMR and ^13^C NMR spectra were recorded on Bruker Avance DRX 500 spectrometer at a resonance frequency of 500 and 126 MHz in DMSO-*d*_6_. Chemical shifts are reported in the δ scale (ppm). Mass spectra were recorded on an Agilent 1100 high-performance liquid chromatograph (HPLC) equipped with a diode matrix and an Agilent LC/MSD SL mass-selective detector, a SUPELCO Ascentis Express C18 chromatographic column 2.7 µm 4.6 mm x 15 cm.

^1^H NMR spectrum, δ, ppm: 10.03 (*s*, 1H), 9.91 (*br. s*, 1H), 8.36 (*d*, *J* = 8.0 Hz, 2H), 8.01 (*d*, *J* = 8.1 Hz, 2H), 7.67 (*d*, *J* = 9.7 Hz, 1H), 7.40–7.24 (*m*, 2H), 3.84 (*s*, 3H) (see Fig. S1 top).

^13^C NMR spectrum, δ, ppm: 193.1, 173.2, 156.5, 150.1, 143.7, 140.4, 137.3, 136.6, 129.9, 128.4, 124.4, 122.2, 120.6, 104.3, 56.2 (see Figure S1 bottom).

Mass spectrum, *m*/*z* (*I*_rel_, %): 297.0 [*M* + H]+(100) (see Fig. S2).

## 7. Refinement

Crystal data, data collection and structure refinement details are summarized in Table 2 ▸. H atoms were placed in calculated positions and refined by riding model with *U*_iso_(H) = *nU*_eq_ of the carrier atom (*n* = 1.5 for methyl groups and *n* = 1.2 for other hydrogen atoms).

## Supplementary Material

Crystal structure: contains datablock(s) I. DOI: 10.1107/S2056989026003476/zn2047sup1.cif

Structure factors: contains datablock(s) I. DOI: 10.1107/S2056989026003476/zn2047Isup2.hkl

Figure S1.1H NMR (top) and 13C NMR (bottom) spectra of 1. DOI: 10.1107/S2056989026003476/zn2047sup3.png

Figure S2. Mass spectrum of 1. DOI: 10.1107/S2056989026003476/zn2047sup4.png

Supporting information file. DOI: 10.1107/S2056989026003476/zn2047Isup5.cml

CCDC reference: 2543411

Additional supporting information:  crystallographic information; 3D view; checkCIF report

## Figures and Tables

**Table 1:** Hydrogen-bond geometry (Å, °)

*D*—H⋯*A*	*D*—H	H⋯*A*	*D*⋯*A*	*D*—H⋯*A*
O3—H3⋯O1^i^	0.89 (2)	2.02 (2)	2.8304 (18)	151 (2)
O3—H3⋯O4	0.89 (2)	2.20 (2)	2.6853 (18)	113.6 (19)
C6—H6⋯O3	0.93	2.20	2.834 (2)	12

Symmetry code: (i) 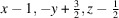.

**Table 2:** Experimental details

Crystal data	
Chemical formula	C_17_H_12_O_5_
*M*_r_	296.27
Crystal system, space group	Monoclinic, *P*2_1_/*c*
Temperature (K)	296
*a*, *b*, *c* (Å)	9.9231 (10), 18.3524 (18), 7.5479 (8)
β (°)	103.118 (3)
*V* (Å^3^)	1338.7 (2)
*Z*	4
Radiation type	Mo *K*α
μ (mm^−1^)	0.11
Crystal size (mm)	0.3 × 0.2 × 0.1

Data collection	
Diffractometer	Bruker APEXII CCD
Absorption correction	Multi-scan (*SADABS*; Krause *et al.*, 2015 ▸)
*T*_min_, *T*_max_	0.652, 0.746
No. of measured, independent and observed [*I* > 2σ(*I*)] reflections	23026, 3062, 2019
*R*_int_	0.063
(sin θ/λ)_max_ (Å^−1^)	0.650

Refinement	
*R*[*F*^2^ > 2σ(*F*^2^)], *wR*(*F*^2^), *S*	0.050, 0.125, 1.04
No. of reflections	3062
No. of parameters	203
H-atom treatment	H atoms treated by a mixture of independent and constrained refinement
Δρ_max_, Δρ_min_ (e Å^−3^)	0.23, −0.21

Computer programs: *APEX2* and *SAINT* (Bruker, 2014 ▸), *SHELXT2018/2* (Sheldrick, 2015*a* ▸), *SHELXL2019/3* (Sheldrick, 2015*b* ▸) and *OLEX2* (Dolomanov *et al.*, 2009 ▸).

## supplementary crystallographic information

### 4-(3-Hydroxy-6-methoxy-4-oxo-4H-chromen-2-yl)benzaldehyde Crystal data

**Table:**

C_17_H_12_O_5_	*F*(000) = 616
*M**_r_* = 296.27	*D*_x_ = 1.470 Mg m^−^^3^
Monoclinic, *P*2_1_/*c*	Mo *K*α radiation, λ = 0.71073 Å
*a* = 9.9231 (10) Å	Cell parameters from 3272 reflections
*b* = 18.3524 (18) Å	θ = 2.2–24.7°
*c* = 7.5479 (8) Å	µ = 0.11 mm^−^^1^
β = 103.118 (3)°	*T* = 296 K
*V* = 1338.7 (2) Å^3^	Block, yellow
*Z* = 4	0.3 × 0.2 × 0.1 mm

### 4-(3-Hydroxy-6-methoxy-4-oxo-4H-chromen-2-yl)benzaldehyde Data collection

**Table:**

Bruker APEXII CCD diffractometer	2019 reflections with *I* > 2σ(*I*)
Graphite monochromator	*R*_int_ = 0.063
φ and ω scans	θ_max_ = 27.5°, θ_min_ = 2.1°
Absorption correction: multi-scan (SADABS; Krause *et al.*, 2015)	*h* = −12→12
*T*_min_ = 0.652, *T*_max_ = 0.746	*k* = −23→23
23026 measured reflections	*l* = −9→9
3062 independent reflections	

### 4-(3-Hydroxy-6-methoxy-4-oxo-4H-chromen-2-yl)benzaldehyde Refinement

**Table:**

Refinement on *F*^2^	Primary atom site location: dual
Least-squares matrix: full	Hydrogen site location: mixed
*R*[*F*^2^ > 2σ(*F*^2^)] = 0.050	H atoms treated by a mixture of independent and constrained refinement
*wR*(*F*^2^) = 0.125	*w* = 1/[σ^2^(*F*_o_^2^) + (0.0524*P*)^2^ + 0.2246*P*] where *P* = (*F*_o_^2^ + 2*F*_c_^2^)/3
*S* = 1.04	(Δ/σ)_max_ < 0.001
3062 reflections	Δρ_max_ = 0.23 e Å^−^^3^
203 parameters	Δρ_min_ = −0.21 e Å^−^^3^
0 restraints	

### 4-(3-Hydroxy-6-methoxy-4-oxo-4H-chromen-2-yl)benzaldehyde Special details

**Table:**

Geometry. All esds (except the esd in the dihedral angle between two l.s. planes) are estimated using the full covariance matrix. The cell esds are taken into account individually in the estimation of esds in distances, angles and torsion angles; correlations between esds in cell parameters are only used when they are defined by crystal symmetry. An approximate (isotropic) treatment of cell esds is used for estimating esds involving l.s. planes.
Refinement. Using Olex2 (Dolomanov *et al.*, 2009), the structure was solved with the SHELXT (Sheldrick, 2018) structure solution program using Intrinsic Phasing and refined with the SHELXL (Sheldrick, 2015) refinement package. Full-matrix least-squares refinement against F^2^ in anisotropic approximation was used for non-hydrogen atoms.

### 4-(3-Hydroxy-6-methoxy-4-oxo-4H-chromen-2-yl)benzaldehyde Fractional atomic coordinates and isotropic or equivalent isotropic displacement parameters (Å^2^)

**Table:**

	*x*	*y*	*z*	*U*_iso_*/*U*_eq_	
O1	1.29775 (13)	0.69700 (7)	0.9257 (2)	0.0517 (4)	
O2	0.64574 (11)	0.53802 (6)	0.76175 (16)	0.0363 (3)	
O3	0.52689 (14)	0.70937 (7)	0.5462 (2)	0.0507 (4)	
H3	0.440 (3)	0.7237 (13)	0.506 (3)	0.076*	
O4	0.28735 (13)	0.63680 (7)	0.5116 (2)	0.0481 (4)	
O5	0.17061 (13)	0.38516 (7)	0.75935 (19)	0.0459 (4)	
C1	1.20045 (19)	0.71828 (10)	0.8100 (3)	0.0397 (5)	
H1	1.215242	0.757103	0.737517	0.048*	
C2	1.06098 (18)	0.68715 (9)	0.7761 (2)	0.0338 (4)	
C3	1.03239 (18)	0.62416 (10)	0.8636 (3)	0.0374 (4)	
H3A	1.103690	0.599247	0.941261	0.045*	
C4	0.89871 (18)	0.59871 (10)	0.8353 (2)	0.0375 (4)	
H4	0.880862	0.556435	0.893953	0.045*	
C5	0.78883 (17)	0.63530 (9)	0.7197 (2)	0.0327 (4)	
C6	0.81938 (19)	0.69798 (10)	0.6320 (3)	0.0402 (5)	
H6	0.748651	0.723149	0.553968	0.048*	
C7	0.95327 (18)	0.72287 (10)	0.6599 (3)	0.0380 (4)	
H7	0.971821	0.764552	0.599509	0.046*	
C8	0.64731 (17)	0.60722 (9)	0.6925 (2)	0.0331 (4)	
C9	0.52761 (18)	0.64075 (9)	0.6130 (2)	0.0353 (4)	
C10	0.39395 (18)	0.60553 (10)	0.5922 (2)	0.0352 (4)	
C11	0.39709 (17)	0.53381 (9)	0.6732 (2)	0.0312 (4)	
C12	0.52311 (17)	0.50293 (10)	0.7537 (2)	0.0326 (4)	
C13	0.53046 (19)	0.43401 (10)	0.8322 (2)	0.0392 (4)	
H13	0.615784	0.413493	0.885235	0.047*	
C14	0.41102 (19)	0.39688 (10)	0.8306 (3)	0.0404 (5)	
H14	0.415206	0.350797	0.882787	0.048*	
C15	0.28218 (18)	0.42748 (10)	0.7510 (2)	0.0354 (4)	
C16	0.27491 (18)	0.49510 (10)	0.6721 (2)	0.0361 (4)	
H16	0.189425	0.515213	0.618100	0.043*	
C17	0.03785 (19)	0.41217 (11)	0.6707 (3)	0.0479 (5)	
H17A	0.035499	0.420573	0.544548	0.072*	
H17B	0.020241	0.457065	0.726766	0.072*	
H17C	−0.031685	0.377089	0.681216	0.072*	

### 4-(3-Hydroxy-6-methoxy-4-oxo-4H-chromen-2-yl)benzaldehyde Atomic displacement parameters (Å^2^)

**Table:**

	*U*^11^	*U*^22^	*U*^33^	*U*^12^	*U*^13^	*U*^23^
O1	0.0366 (8)	0.0462 (9)	0.0653 (10)	−0.0042 (6)	−0.0031 (7)	0.0009 (7)
O2	0.0305 (7)	0.0323 (7)	0.0453 (8)	0.0010 (5)	0.0071 (5)	0.0060 (6)
O3	0.0325 (7)	0.0322 (8)	0.0835 (11)	0.0029 (6)	0.0051 (7)	0.0152 (7)
O4	0.0315 (7)	0.0401 (8)	0.0685 (10)	0.0041 (6)	0.0027 (6)	0.0121 (7)
O5	0.0385 (8)	0.0413 (8)	0.0565 (9)	−0.0074 (6)	0.0077 (6)	0.0086 (6)
C1	0.0383 (10)	0.0371 (11)	0.0443 (12)	−0.0015 (8)	0.0103 (8)	−0.0043 (8)
C2	0.0337 (10)	0.0303 (10)	0.0380 (10)	−0.0001 (8)	0.0097 (7)	−0.0062 (8)
C3	0.0322 (10)	0.0352 (10)	0.0418 (11)	0.0037 (8)	0.0024 (8)	0.0009 (8)
C4	0.0376 (10)	0.0316 (10)	0.0430 (11)	0.0017 (8)	0.0087 (8)	0.0025 (8)
C5	0.0305 (9)	0.0316 (10)	0.0359 (10)	0.0038 (7)	0.0073 (7)	−0.0033 (7)
C6	0.0339 (10)	0.0374 (11)	0.0486 (12)	0.0040 (8)	0.0081 (8)	0.0085 (9)
C7	0.0370 (10)	0.0331 (10)	0.0443 (11)	−0.0001 (8)	0.0105 (8)	0.0046 (8)
C8	0.0335 (10)	0.0289 (10)	0.0372 (10)	0.0016 (7)	0.0087 (7)	0.0007 (7)
C9	0.0354 (10)	0.0270 (10)	0.0431 (11)	0.0010 (8)	0.0082 (8)	0.0018 (8)
C10	0.0310 (10)	0.0341 (10)	0.0399 (11)	0.0024 (8)	0.0066 (8)	−0.0005 (8)
C11	0.0307 (9)	0.0294 (9)	0.0338 (10)	0.0011 (7)	0.0081 (7)	−0.0009 (7)
C12	0.0323 (9)	0.0313 (10)	0.0342 (10)	0.0004 (7)	0.0075 (7)	−0.0009 (7)
C13	0.0379 (10)	0.0360 (11)	0.0415 (11)	0.0049 (8)	0.0044 (8)	0.0062 (8)
C14	0.0436 (11)	0.0333 (10)	0.0427 (11)	−0.0001 (8)	0.0066 (8)	0.0068 (8)
C15	0.0372 (10)	0.0345 (10)	0.0352 (10)	−0.0051 (8)	0.0096 (8)	−0.0023 (8)
C16	0.0329 (10)	0.0352 (10)	0.0398 (11)	0.0012 (8)	0.0071 (7)	−0.0003 (8)
C17	0.0358 (11)	0.0466 (12)	0.0604 (14)	−0.0061 (9)	0.0093 (9)	0.0029 (10)

### 4-(3-Hydroxy-6-methoxy-4-oxo-4H-chromen-2-yl)benzaldehyde Geometric parameters (Å, º)

**Table:**

O1—C1	1.210 (2)	C6—H6	0.9300
O2—C8	1.375 (2)	C6—C7	1.375 (2)
O2—C12	1.366 (2)	C7—H7	0.9300
O3—H3	0.89 (2)	C8—C9	1.351 (2)
O3—C9	1.356 (2)	C9—C10	1.451 (2)
O4—C10	1.2354 (19)	C10—C11	1.449 (2)
O5—C15	1.366 (2)	C11—C12	1.382 (2)
O5—C17	1.424 (2)	C11—C16	1.404 (2)
C1—H1	0.9300	C12—C13	1.392 (2)
C1—C2	1.465 (2)	C13—H13	0.9300
C2—C3	1.392 (2)	C13—C14	1.365 (2)
C2—C7	1.384 (2)	C14—H14	0.9300
C3—H3A	0.9300	C14—C15	1.401 (2)
C3—C4	1.377 (2)	C15—C16	1.371 (3)
C4—H4	0.9300	C16—H16	0.9300
C4—C5	1.403 (2)	C17—H17A	0.9600
C5—C6	1.395 (3)	C17—H17B	0.9600
C5—C8	1.466 (2)	C17—H17C	0.9600
			
C12—O2—C8	120.37 (13)	C8—C9—C10	122.34 (16)
C9—O3—H3	108.8 (16)	O4—C10—C9	120.39 (16)
C15—O5—C17	116.97 (14)	O4—C10—C11	124.31 (16)
O1—C1—H1	117.7	C11—C10—C9	115.29 (15)
O1—C1—C2	124.56 (18)	C12—C11—C10	119.27 (15)
C2—C1—H1	117.7	C12—C11—C16	119.29 (16)
C3—C2—C1	121.82 (17)	C16—C11—C10	121.44 (15)
C7—C2—C1	119.26 (17)	O2—C12—C11	122.29 (15)
C7—C2—C3	118.86 (16)	O2—C12—C13	116.72 (15)
C2—C3—H3A	120.0	C11—C12—C13	120.99 (16)
C4—C3—C2	120.07 (17)	C12—C13—H13	120.4
C4—C3—H3A	120.0	C14—C13—C12	119.23 (17)
C3—C4—H4	119.3	C14—C13—H13	120.4
C3—C4—C5	121.30 (17)	C13—C14—H14	119.7
C5—C4—H4	119.3	C13—C14—C15	120.67 (17)
C4—C5—C8	120.21 (16)	C15—C14—H14	119.7
C6—C5—C4	117.91 (16)	O5—C15—C14	115.02 (16)
C6—C5—C8	121.88 (16)	O5—C15—C16	124.85 (16)
C5—C6—H6	119.7	C16—C15—C14	120.13 (17)
C7—C6—C5	120.51 (17)	C11—C16—H16	120.2
C7—C6—H6	119.7	C15—C16—C11	119.69 (17)
C2—C7—H7	119.3	C15—C16—H16	120.2
C6—C7—C2	121.33 (17)	O5—C17—H17A	109.5
C6—C7—H7	119.3	O5—C17—H17B	109.5
O2—C8—C5	111.39 (14)	O5—C17—H17C	109.5
C9—C8—O2	120.31 (15)	H17A—C17—H17B	109.5
C9—C8—C5	128.29 (16)	H17A—C17—H17C	109.5
O3—C9—C10	116.47 (15)	H17B—C17—H17C	109.5
C8—C9—O3	121.19 (16)		
			
O1—C1—C2—C3	−7.2 (3)	C7—C2—C3—C4	−0.6 (3)
O1—C1—C2—C7	170.05 (18)	C8—O2—C12—C11	1.6 (2)
O2—C8—C9—O3	179.08 (16)	C8—O2—C12—C13	−177.93 (16)
O2—C8—C9—C10	−1.7 (3)	C8—C5—C6—C7	−179.85 (17)
O2—C12—C13—C14	179.03 (16)	C8—C9—C10—O4	−176.81 (18)
O3—C9—C10—O4	2.4 (3)	C8—C9—C10—C11	3.9 (3)
O3—C9—C10—C11	−176.89 (16)	C9—C10—C11—C12	−3.3 (2)
O4—C10—C11—C12	177.41 (18)	C9—C10—C11—C16	176.49 (16)
O4—C10—C11—C16	−2.8 (3)	C10—C11—C12—O2	0.7 (3)
O5—C15—C16—C11	179.50 (15)	C10—C11—C12—C13	−179.71 (17)
C1—C2—C3—C4	176.63 (16)	C10—C11—C16—C15	−179.66 (17)
C1—C2—C7—C6	−176.28 (17)	C11—C12—C13—C14	−0.5 (3)
C2—C3—C4—C5	−0.3 (3)	C12—O2—C8—C5	178.44 (14)
C3—C2—C7—C6	1.0 (3)	C12—O2—C8—C9	−1.1 (2)
C3—C4—C5—C6	0.8 (3)	C12—C11—C16—C15	0.1 (3)
C3—C4—C5—C8	−179.71 (16)	C12—C13—C14—C15	0.0 (3)
C4—C5—C6—C7	−0.4 (3)	C13—C14—C15—O5	−179.54 (16)
C4—C5—C8—O2	−11.8 (2)	C13—C14—C15—C16	0.6 (3)
C4—C5—C8—C9	167.75 (18)	C14—C15—C16—C11	−0.7 (3)
C5—C6—C7—C2	−0.5 (3)	C16—C11—C12—O2	−179.07 (15)
C5—C8—C9—O3	−0.4 (3)	C16—C11—C12—C13	0.5 (3)
C5—C8—C9—C10	178.76 (16)	C17—O5—C15—C14	−176.38 (16)
C6—C5—C8—O2	167.64 (16)	C17—O5—C15—C16	3.4 (3)
C6—C5—C8—C9	−12.8 (3)		

### 4-(3-Hydroxy-6-methoxy-4-oxo-4H-chromen-2-yl)benzaldehyde Hydrogen-bond geometry (Å, º)

**Table:**

*D*—H···*A*	*D*—H	H···*A*	*D*···*A*	*D*—H···*A*
O3—H3···O1^i^	0.89 (2)	2.02 (2)	2.8304 (18)	151 (2)
O3—H3···O4	0.89 (2)	2.20 (2)	2.6853 (18)	113.6 (19)
C6—H6···O3	0.93	2.20	2.834 (2)	12

Symmetry code: (i) *x*−1, −*y*+3/2, *z*−1/2.
