# Supplementary figures and images for: Synthesis, crystal structure and Hirshfeld surface analysis of 4-(3-hy­droxy-6-meth­oxy-4-oxo-4H-chromen-2-yl)benzaldehyde
Source: Acta Crystallogr E Crystallogr Commun. 2026 Apr 10;82(Pt 5):459–62. doi: 10.1107/S2056989026003476 (PMC13148196; doi:10.1107/S2056989026003476)

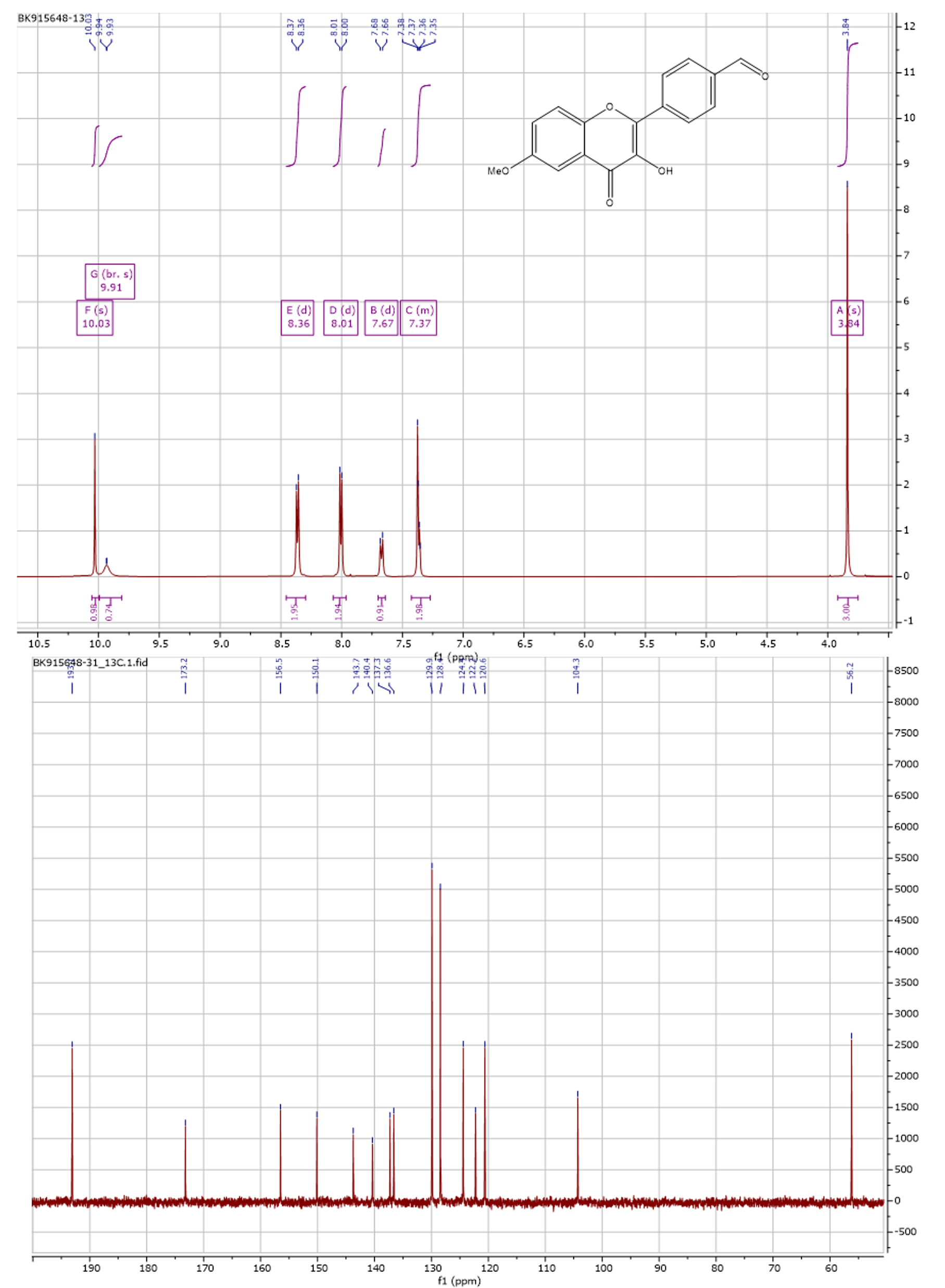

Supplement: Supplementary file 3 [file e-82-00459-sup3.png]

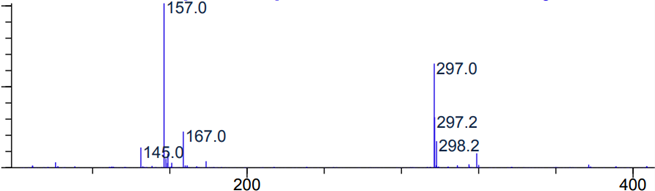

Supplement: Supplementary file 4 [file e-82-00459-sup4.png]
